# Leprosy and Its Prevention

**Published:** 1890-03

**Authors:** 


*Leprosy and its Prevention.* By ROBSON ROOSE, M.D., LL.D. Pp. 100. London: H. K. LEWIS. 1890.

The title of this work is more pretentious than its contents. Only under very exceptional circumstances could "several visits to Norway" justify a physician, with no other opportunities for clinical study, in writing a book on "Leprosy and its Prevention;" these circumstances are not very evident in this case, and the result is as might be expected. Very few men in England are competent to write or to review a work on Leprosy; and neither the author of this work nor his critic is among the number.

